# Linear and Non-linear Dimensionality-Reduction Techniques on Full Hand Kinematics

**DOI:** 10.3389/fbioe.2020.00429

**Published:** 2020-05-05

**Authors:** Alexandra A. Portnova-Fahreeva, Fabio Rizzoglio, Ilana Nisky, Maura Casadio, Ferdinando A. Mussa-Ivaldi, Eric Rombokas

**Affiliations:** ^1^Department of Mechanical Engineering, Northwestern University, Evanston, IL, United States; ^2^Shirley Ryan Ability Lab, Chicago, IL, United States; ^3^Department of Physiology, Feinberg School of Medicine, Northwestern University, Chicago, IL, United States; ^4^Department of Informatics, Bioengineering, Robotics and Systems Engineering, University of Genoa, Genoa, Italy; ^5^Department of Biomedical Engineering, Ben-Gurion University of the Negev, Be'er Sheva, Israel; ^6^Department of Mechanical Engineering, University of Washington, Seattle, WA, United States; ^7^Department of Electrical Engineering, University of Washington, Seattle, WA, United States

**Keywords:** kinematics, neural networks, principal component analysis, dimensionality reduction, unsupervised learning, prosthetics

## Abstract

The purpose of this study was to find a parsimonious representation of hand kinematics data that could facilitate prosthetic hand control. Principal Component Analysis (PCA) and a non-linear Autoencoder Network (nAEN) were compared in their effectiveness at capturing the essential characteristics of a wide spectrum of hand gestures and actions. Performance of the two methods was compared on (a) the ability to accurately reconstruct hand kinematic data from a latent manifold of reduced dimension, (b) variance distribution across latent dimensions, and (c) the separability of hand movements in compressed and reconstructed representations derived using a linear classifier. The nAEN exhibited higher performance than PCA in its ability to more accurately reconstruct hand kinematic data from a latent manifold of reduced dimension. Whereas, for two dimensions in the latent manifold, PCA was able to account for 78% of input data variance, nAEN accounted for 94%. In addition, the nAEN latent manifold was spanned by coordinates with more uniform share of signal variance compared to PCA. Lastly, the nAEN was able to produce a manifold of more separable movements than PCA, as different tasks, when reconstructed, were more distinguishable by a linear classifier, SoftMax regression. It is concluded that non-linear dimensionality reduction may offer a more effective platform than linear methods to control prosthetic hands.

## Introduction

The complexity of the human hand makes it the subject of intensive research in prosthetics and robotics control. Controlling several degrees of freedom (DOFs)—there are 27 in each hand—can be a difficult task when both precision and speed are required as in dexterous prosthetic hand control. Since their first development in the 1940s, myoelectric prostheses, operated by electromyographic (EMG) signals, have undergone a series of design and control changes (Zuo and Olson, 2014).

Technological advances have resulted in more complex prostheses with an increased number of DOFs (Belter et al., 2013). The increase in design complexity was also associated with the high demand of prosthetic users to be able to perform dexterous tasks, such as handicrafts, operation of domestic and electronic devices, as well as dressing/undressing (Pylatiuk et al., 2007). For acceptable performance in such tasks, individual digit control is often required. Instead of allowing the independent control of each degree of freedom, currently available market options include a variety of prosthetic hands with a limited number of preset gestures associated with the most common grasp patterns to be performed in activities of daily living (ADLs). For example, the Michelangelo Hand (*Ottobock, Duderstadt, Germany*) includes seven grip patterns whereas its successor, the Bebionic Hand, from the same company includes 14 grip patterns.

While there have been many developments in the design of prosthetic hands, advances in control strategies have been limited. There are numerous types of controls used in upper-limb myoelectric prostheses from simple finite-state machines (FSM) to complex pattern recognition (PR) (Geethanjali, 2016). In FSM, usually two EMG signals are used to switch between grip patterns. This method can be effective for a small number of postures but in the case of 14 or even seven grips, this can be a strenuous and time-consuming task. In addition to slow controller speeds, prosthetic users have identified their myoelectric device speeds as inadequate for task completion (Pylatiuk et al., 2007). Such issues in the device performance could be contributing to the high abandonment rates that are prevalent in upper-limb prostheses (Biddiss and Chau, 2007).

Recently, a new type of prosthetic control has been proposed—*posture control* (Geethanjali, 2016). This capitalizes on the results of previous dimensionality-reduction studies performed on hand kinematics (Santello et al., 1998; Todorov and Ghahramani's, 2004; Ingram et al., 2008). In these studies, Principal Component Analysis (PCA) was utilized to simplify the complex kinematics of hand grasps by finding a reduced number of linear combinations of input signals that explain most of the variability observed in grasping data. These combinations span the latent manifold of hand kinematics. By projecting the data along these directions, it is possible to obtain a compressed representation of hand configurations. A related approach to the control of prosthetic hands was introduced by Bicchi's group with the “Soft hand” (Della Santina et al., 2017, 2018). The underlying novel idea is that a variety of grasping patterns can be obtained by a single “synergy” of compliant actuators interacting mechanically with different shapes of grasped objects. While we recognize the value of this approach for the simplification of hand control during manipulation, here we are also considering the value of the hand as a communication device. In this broader scope, as well as in the performance with musical instruments, the ability to explicitly and precisely control finger configuration is essential. For this, we are considering in this study a data set obtained from American Sign Language (ASL).

In some myoelectric control methods, EMG signals are mapped to control parameters of the latent manifold, or principal components (PCs), which are derived through PCA (Matrone et al., 2010, 2012; Segil, 2013, 2015; Segil and Huddle, 2016). An important difference of this method with respect to FSM and PR control is that posture control is continuous rather than discrete. This allows users to directly operate the prosthetic device in a way more consistent with natural movement control instead of being limited to a finite set of pre-defined grasp options.

While PCA is a computationally straightforward and inexpensive procedure, it is limited by its ability to only account for linear relationships in the input signals. The assumption of linearity is not consistent with the geometry of hand kinematics.

To account for kinematic non-linearities, this study considers a dimensionality-reduction method based on autoencoder networks (AENs). AENs are artificial neural networks that are trained to reconstruct their inputs. They are composed of two parts: an encoder that converts the input data to a lower-dimensional, *latent*, manifold and a decoder that converts the latent manifold into the outputs. AENs provide an unsupervised method, reconstructing inputs in their outputs, without the requirement of labeled data. Most importantly, AENs are able to cope with both linear and non-linear relations in the input data by making use of linear and non-linear activation functions.

This study compares the performance of a non-linear AEN (nAEN) to that of PCA on examples of hand kinematics observed in human participants. In addition, it evaluates the case for the potential use of nAENs over PCA in a prosthetic controller.

## Materials and Methods

### Experimental Setup

Ten unimpaired right-handed individuals (six males, four females, 32.8 ± 9.4 years old) participated in this study. Participant recruitment and data collection conformed with the University of Washington's Institutional Review Board (IRB). Informed written consent was obtained from each participant. Basic measurements were taken from the right hand of each participant and recorded with other information.

**Figure 1:**
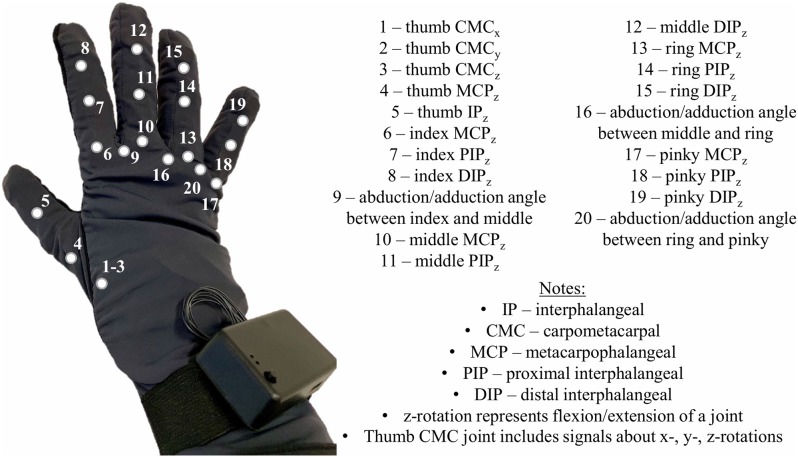
Virtual Motion Labs Glove used for the study to record kinematics of the right hand. Numbers on the glove represent the kinematic signals that were extracted and recorded for analysis.

The participants were first fitted with a right-handed data glove (*Virtual Motion Labs, Dallas, TX, USA*). A total of 20 signals were extracted from the glove that accounted for finger joint kinematics (Figure 1). The signals were recorded at a sampling rate of 100 Hz.

To calibrate the data glove, the participants were asked to perform a series of hand gestures presented to them on the screen. The movements were finger flexion/extension, finger abduction/adduction, thumb flexion/extension, and individual finger flexion against the thumb.

**Figure 2:**
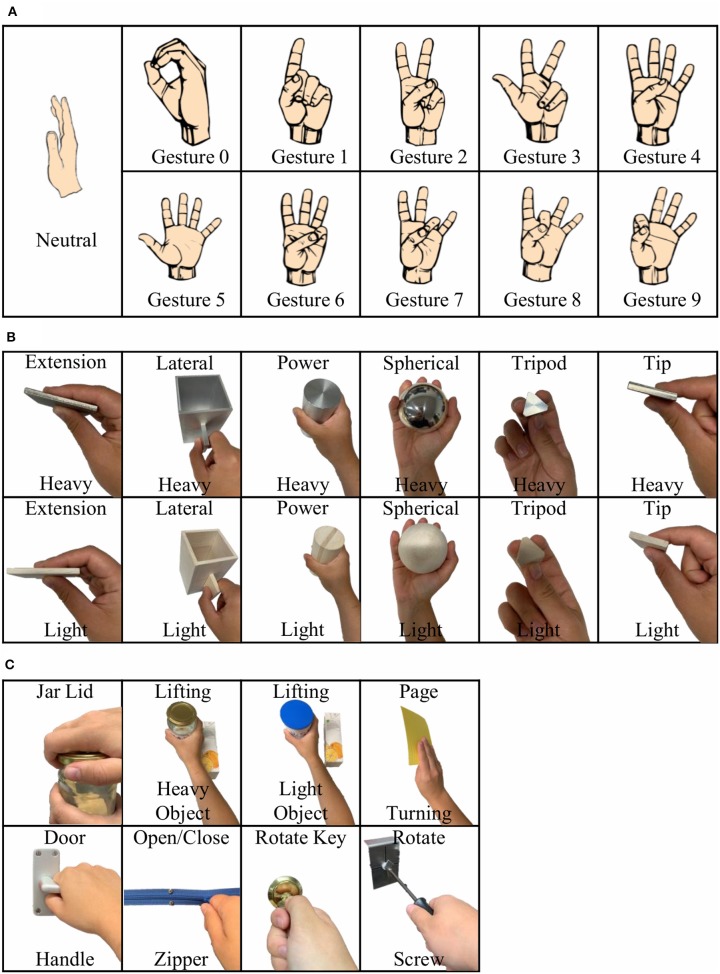
Study setup consisting of three different phases: **(A)** American Sign Language (ASL) Gestures; **(B)** Object Grasps; **(C)** Activities of Daily Living (ADL) Tasks.

Once the glove was calibrated, the participants were guided through a sequence of hand movements consisting of: (i) ASL Gestures, (ii) Object Grasps, and (iii) ADL Tasks (Figure 2) as described in the following.

#### ASL Gestures

During the first phase of the experiment, the participants were asked to perform 10 different ASL gestures (Figure 2A). They were asked to repeat each gesture 10 times. Each gesture repetition counted as a trial. At the beginning of each trial, the participants started in the REST position with their elbows on the table and the right hand raised straight up.

When presented with a gesture, the participants were given 3 s to mimic it as displayed on the screen and maintain the gesture until instructed to return to REST position. After 3 s in the REST position, a new trial would start. The participants had an opportunity to practice the gestures before the beginning of the phase.

#### Object Grasps

During the second phase of the experiment, the participants were asked to perform a series of object grasps from the Southampton Hand Assessment Procedure (SHAP) (Figure 2B) (Light et al., 2002). The testing board was placed 8 cm from the edge of the table closest to the participant. The board was then aligned so that the target object was directly in front of the participant. There were 12 objects to be grasped with six different grasping types. Furthermore, each object could be either light or heavy.

Each object had to be grasped 10 times. Before grasping a new object, video instructions were shown to the participants on the required way of grasping. They could then practice grasping under the supervision of the experimenter to ensure a correct and consistent execution.

The participants were given 5 s to complete each grasp starting and ending the grasp on the REST position. In the REST position, both participant's hands lied prone on the table. Between each trial, there was a 5 s resting period.

#### ADL Tasks

During the third phase of the experiment, the participants performed the ADL portion of the SHAP. A total of eight different tasks were selected for this phase (Figure 2C). Each task was performed 10 times. The participants were given 7 s to perform each task with a 5 s of REST time between each trial.

As in the second phase, the participants were shown a video with instructions on how to appropriately complete the task. They were then instructed to practice the task until ready. The testing board was placed 8 cm away from the edge of the table closest to the participant. During REST, the participants held the hands supine on the table to the sides of the testing board.

### Data Processing

For each phase, data were recorded during both REST (when participants were instructed to be in REST position) and ACTIVE (when participants were instructed to perform the given task) conditions. Only ACTIVE conditions were used for data analysis.

#### Preprocessing

The recorded data were filtered with a first-order Butterworth filter in *MATLAB* (*MathWorks, Natick, MA, USA*). The cutoff frequency was 10 Hz. REST data were removed from analysis, and the remaining data points were labeled to indicate different hand movements and trial numbers for each participant.

Trials, in which participants did not complete the movement as requested, possibly due to loss of attention or inability to understand the given task in due time, were excluded from the analysis.

The data were then arranged into four datasets: *ASL Gestures, Object Grasps, ADL Tasks*, and *Combined*. The *Combined* dataset contained data from *ASL Gestures, Object Grasps*, and *ADL Tasks* for each participant.

Each signal was normalized by the absolute maximum value across all signals in each dataset prior to analysis (Sola and Sevilla, 1997).

Data of each participant were randomly split into training (80%) and testing (20%) using a holdout method (Oxford and Daniel, 2001). Training samples were used to generate a model, PCA or nAEN.

### Data Analysis

To study the effects of linear and non-linear dimensionality-reduction methods, we compared the performance of PCA and nAEN algorithms with two, three, four, five, and six latent dimensions [PCs and coding units (CUs), respectively].

PCA was performed using the built-in *MATLAB* function *pca*, which is based on singular value decomposition (Madsen et al., 2004). Dimensionality reduction using nAEN was performed using *TensorFlow*, a Python *(Python Software Foundation, DE, USA)* library for machine learning applications developed by Google Brain (Abadi et al., 2016).

#### nAEN Architecture

**Figure 3:**
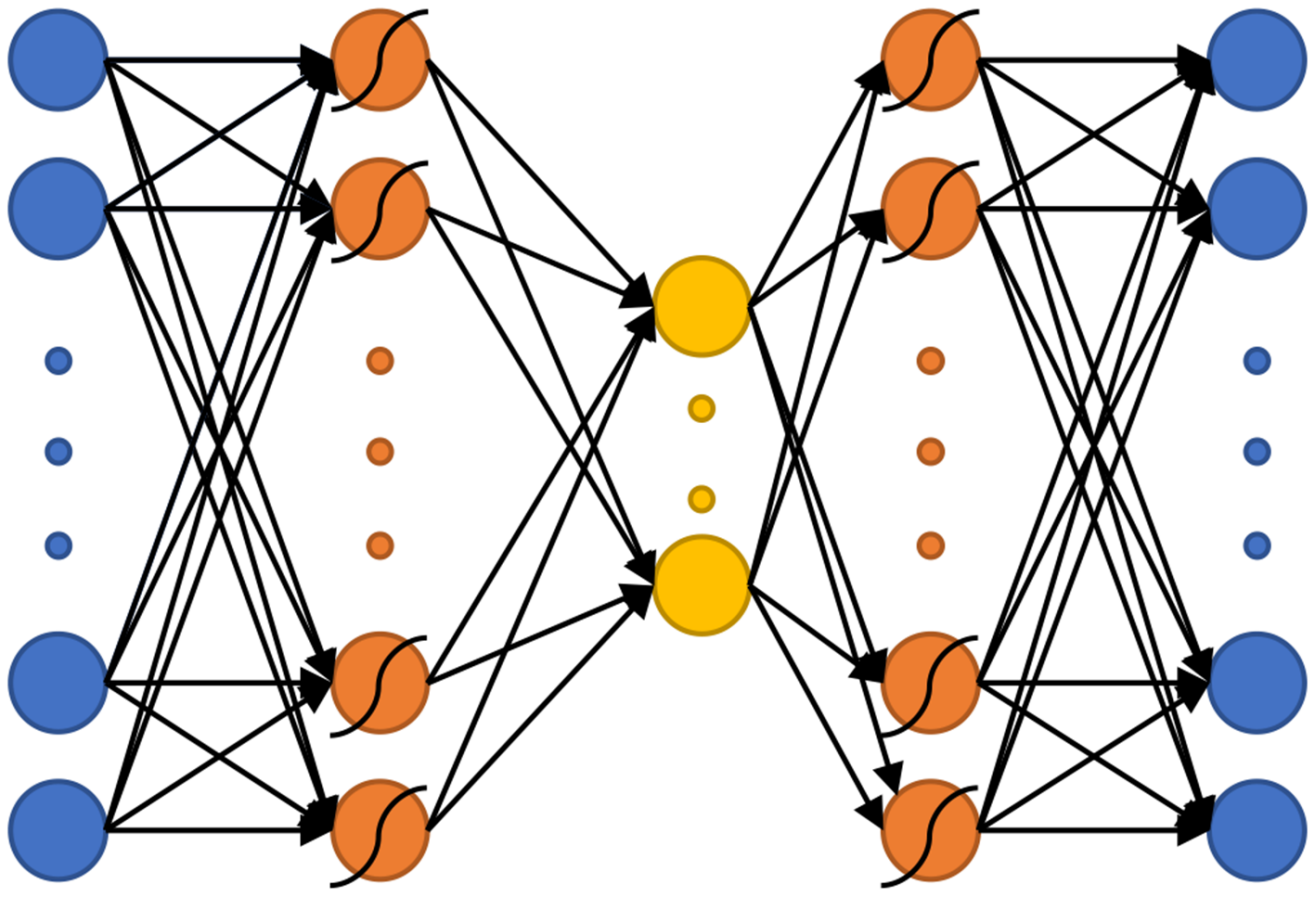
The non-linear Autoencoder Network (nAEN) structure used in this study. Curved lines over neurons represent that non-linear activation functions (i.e., hyperbolic tangent) were used to calculate that layer. Otherwise, activation functions were linear.

The basic nAEN structure used for this experiment included a total of three hidden layers, the middle one being the bottleneck layer (Figure 3). Similarly to the original AEN proposed by Kramer (1991), we chose a non-linear activation function for the first and third hidden layers, and a linear activation function for the bottleneck (Kramer, 1991). The use of both linear and non-linear activation functions had been shown to increase the ease with which the network learns linear relationships in the data (Haesloop and Holt, 1990). The transformations from the normalized input *X* to the output data *Y* through the *encoder* (Equations 1, 2) and *decoder* (Equations 3, 4) parts of nAEN are:

(1) $$\begin{array}{rcl} layer_{1} & = & \text{tan}h\;\left(X\;*\;w_{1}+b_{1}\right) \end{array}$$

(2) $$\begin{array}{rcl} layer_{2} & = & layer_{1}\;*\;w_{2}+b_{2} \end{array}$$

(3) $$\begin{array}{rcl} layer_{3} & = & \text{tan}h\;\left(layer_{2}\;*\;w_{3}+b_{3}\right) \end{array}$$

(4) $$\begin{array}{rcl} Y & = & layer_{3}\;*\;w_{4}+b_{4} \end{array}$$

where *w*_*i*_ were the weights and *b*_*i*_ were the biases found during network modeling.

While the main purpose of the study was to compare the performance of linear and non-linear dimensionality-reduction algorithms, it is important to note that neural network structures, such as AENs, can be optimized further for improved performance. A simple nAEN structure was chosen for this study while more complex structures can be explored. Neural networks can have multiple layers, various structures, and many hyperparameters that directly affect the performance of the network.

Full-batch gradient descent was used for training the network.

**Figure 4:**
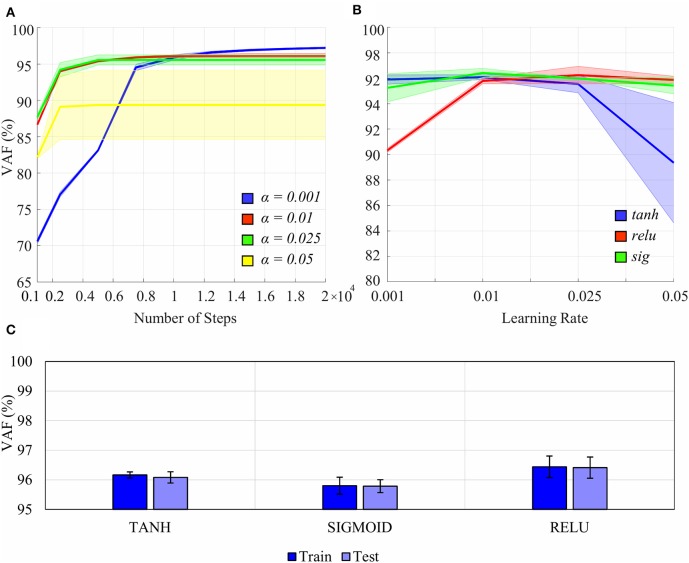
Effects of certain hyperparameters on the non-linear Autoencoder Network (nAEN) performance: **(A)** number of steps and learning rate (0.001, 0.01, 0.025, 0.05); **(B)** learning rate and type of non-linear activation function (*tanh, relu, sig*). The performance is calculated using Variance Accounted For (VAF), which represents the difference between the output and the input. Solid lines represent average results across all paritcipants. Faint shaded lines represent 95% confidence interval. **(C)** nAEN performance difference on training and testing datasest for different activation functions with no regularization. Error bars represent 95% confidence intervals. The nAEN performance difference was minimal across the learning rates tested for over 10,000 steps. The difference in the performance was minimal for the learning rate of 0.01 across the non-linear activation functions tested. No evidence of overfitting of the dataset was found.

Tuning of hyperparameters, such as the learning rate, number of steps, type of non-linear activation functions, and regularization, was performed (Figure 4). A separate validation dataset from a participant, whose data were not used in the experiment for overall analysis, was utilized for hyperparameter tuning. In this dataset, the participant (P0) performed all the tasks of the *ASL Gestures* phase, 10 trials each. A 5-fold cross-validation (CV) was conducted (Oxford and Daniel, 2001), and the performance of each hyperparameter pair was evaluated using Variance Accounted For (VAF) (see section Performance Metrics for more information on VAF).

Learning rates of 0.01 and 0.025 produced the most stable results across the variety of number of steps tested (Figure 4A). The largest learning rate of 0.05 produced the worst results while the smallest learning rate of 0.001 produced the best results for the number of steps over 10,000. The nAEN performance difference was minimal across the learning rates of 0.01, 0.05, and 0.001 for 10,000 steps. Although 20,000 steps resulted in a slightly improved performance of the network for the learning rate of 0.001, a larger number of steps would lead to longer training times for nAEN. Since it was of interest to optimize both the network performance as well as the training times, the learning rate of 0.01 and 10,000 steps were chosen for this study.

Sigmoid (*sig*), hyperbolic tangent (*tanh*), and rectified linear unit (*relu*) performed similarly for the learning rate of 0.01 (Figure 4B). As a result, we were free to choose any non-linear activation function for the nAEN structure, and the hyperbolic tangent was chosen for this experiment.

Adaptive Moment Estimation (ADAM) optimizer was used to speed up the training of the nAEN (Kingma and Ba, 2014). No regularization was used in training the model for this study, as we did not find any evidence of overfitting for our dataset (Figure 4C).

For nAEN, the weights and biases for all models created for each participant were initialized in the same way for comparative purposes.

### Performance Metrics

The performance in terms of VAF was evaluated on the testing samples. Variability of dimensions as well as visualization and separability of movements in the latent and reconstructed manifolds were tested on the entire dataset (training and testing combined).

#### Dimensionality Reduction

VAF measures the difference between reconstructed output and original input signals. It was chosen to capture the ability of a dimensionality-reduction method to reconstruct the desired signal from the latent manifold. VAF offers a measure of the information preserved by the dimensionality-reduction algorithm and is directly related to reconstruction error (Equation 5). A VAF of 100% indicated that the output and the input were identical.

(5) $$\begin{array}{rcl} VAF\left(\%\right) & = & \left(1-\frac{var\left(Y-Ŷ\right)}{var\left(Y\right)}\right)*100 \end{array}$$

$$\begin{array}{rcl} Y & - & original\;data\\ Ŷ & - & reconstructed\;data \end{array}$$

In addition, data variance across three datasets (ASL Gestures, Object Grasps, ADL Tasks) was calculated for each participant. It was done by first calculating the variance of each of the 20 kinematic signals across all samples in the input data. The variance values were then averaged across 20 signals to produce one value of variance for each participant. The correlation between data variance and resulted VAF with two latent dimensions was calculated for both PCA and nAEN.

Lastly, to explore the reconstructing performance of nAEN and PCA, the second gesture of the ASL Gesture dataset (“gesture signifying number one”) was reconstructed from two-dimensional latent manifolds of these dimensionality-reduction methods. To visualize the reconstructed gesture, one of the *Leap Motion* (*Leap Motion Inc., San Francisco, CA, USA*) hand models was utilized in *Unity* (*Unity Technologies, San Francisco, CA, USA*). A few snapshots were taken as the reconstructed gesture went from the REST into the ACTIVE positions.

#### Dimension Variance

Dimension variance was the variance associated with each dimension in the latent manifold of PCA and nAEN. To calculate dimension variance, the input data were reduced to PCs and CUs for each participant across each of the four datasets. For PCA, it was done in the following way, where *eig* produces a diagonal matrix *D* of eigenvalues of a covariance matrix, *cov*, of normalized data *X* (Equation 6).

(6) $$\begin{array}{r} D=eig\left(cov\left(X\right)\right) \end{array}$$

Each PCA dimension, *PC*_*i*_, where *i* was the dimension number, was found by sorting the diagonal matrix *D* in the descending order and taking the *ith* column of the sorted matrix (Equation 7).

(7) $$\begin{array}{r} PC_{i}=diag\left(D\left(:,i\right)\right) \end{array}$$

For nAEN, latent dimensions were calculated by passing the normalized data *X* through the *encoder* part of the network (Equations 1, 2). CUs were the corresponding columns of *layer*_2_ (Equation 8).

(8) $$\begin{array}{r} CU_{i}=layer_{2}\left(:,i\right) \end{array}$$

Each latent dimension (PCs or CUs) was represented by *A*_*i*_, an *m* × *n* matrix, where *m* was the number of observations and *n* was the number of latent dimensions. The mean of each latent dimension was calculated (Equation 9).

(9) $$\begin{array}{r} \mu =\frac{1}{m}\sum_{j=1}^{m}A_{j} \end{array}$$

Afterwards, the variance of each latent dimension, υ_*i*_, was calculated (Equation 10).

(10) $$\begin{array}{r} v_{i}=\frac{1}{m-1}\sum_{j=1}^{m}||A_{j}-\mu |{|}^{2} \end{array}$$

Lastly, what was defined as dimension variance in this study, *v*_dim_, was calculated by determining the percentage of *v*_*i*_ with respect to the overall variance of all considered latent dimensions (Equation 11).

(11) $$\begin{array}{r} v_{\dim}=\frac{v_{i}}{\sum_{i=1}^{n}v_{i}} \end{array}$$

Dimension variance was ordered in descending order for visualization and comparative purposes.

In addition, the average difference between dimensions with the highest and lowest values of variance (*range*_dim, *avg*_) was calculated across all 10 participants for each dataset and called *range of dimension variance* (Equation 12).

(12) $$\begin{array}{r} range_{dim,avg}=\frac{1}{n}\sum_{dim=1}^{n}\max\left(v_{dim}\right)-\min\left(v_{dim}\right). \end{array}$$

#### Latent Trajectories Visualization

To aid the visualization of the latent trajectories, we focused on manifolds with two dimensions. To visualize the PCA latent trajectories, the input data was first reduced to 2PCs. The PC pairs for each dataset and each participant were then plotted on a 2D surface where PC_1_ represented the x-axis and PC_2_ represented the y-axis. Each sample representing a kinematic instance in 20D space was plotted as a point in this 2D graph.

A similar technique was utilized for visualizing the latent trajectories of nAEN. After reducing the input data to the latent manifold, the pair of CUs for each dataset was plotted on a 2D linear surface where CU_1_ represented the x-axis and CU_2_ represented the y-axis.

Separate movements for each dataset were plotted with a different color for ease of differentiation.

#### Movement Separability

Movement separability was defined as a measure of distance between movements (from the 20D input, latent manifold, or reconstructed 20D data) that allowed a simple classification algorithm to differentiate between given classes of postures. Movement separability was calculated for all datasets, except for Combined, using SoftMax regression (Gao and Pavel, 2017). SoftMax regression was chosen as a simple example of a linear classification algorithm that did not require hyperparameter tuning such as Dense Neural Networks (DNNs), which can also be used for classification purposes (Schmidhuber, 2015). The aim was to develop a simple understanding of the linear separability of different movements across latent manifolds of nAEN and PCA. Higher accuracy percentage indicated a latent manifold in which classes (i.e., hand movements) were more linearly separable.

When designing a controller, creating a space where different movements can be easily separated can be of high importance. When navigating along a more separable control space, the user might have the ability to switch between different tasks and/or movements much faster than in cases where tasks are less separable.

SoftMax regression was applied to the 2CUs/PCs latent manifolds, as well as to the reconstructed 20D data for nAEN and PCA and the original input data for each participant and dataset. Assessing the separability of reconstructed space might be important in understanding how data variability is preserved upon reconstruction in both nAEN and PCA. We used a 5-fold CV to calculate the accuracy on each dataset.

### Statistical Analysis

We used MATLAB Statistics Toolbox functions and custom-written code for our statistical analysis. The normality was tested by applying the Anderson-Darling (AD) Test (Anderson and Darling, 1954). When the normality assumption was violated, we used rank statistics for our statistical analysis. This was the case for the VAF, range of dimension variance, and classification accuracy.

We used the Wilcoxon Signed-Rank Test (Wilcoxon, 1945) to understand the differences in VAF between the dimensionality-reduction methods, and the Friedman's Test (Friedman, 1937) to understand the effect of datasets (e.g., ADL Tasks, ASL Gestures, etc.) on VAF. In a *post-hoc* analysis, Bonferroni correction (Bonferroni et al., 1936) was used to verify statistically significant differences among datasets within nAEN and PCA.

We also used the Wilcoxon Signed-Rank Test to compare the differences in range of dimension variance between two dimensionality-reduction methods across all latent dimensions and datasets, and for the comparison of SoftMax classification accuracy between reduced-dimension (2D) and reconstructed (20D) data for both nAEN and PCA.

In all our analyses, the level of significance was set to 0.05. After the Bonferroni adjustment, the level of significance for the *post-hoc* analysis was set to 0.0125 (0.05/4).

## Results

### Dimensionality Reduction

**Figure 5:**
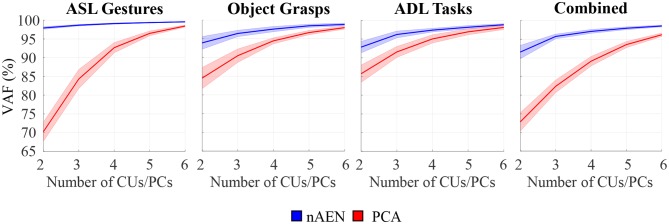
Performance of non-linear Autoencoder Network (nAEN, blue) vs. Principal-Component Analysis (PCA, red) with 2, 3, 4, 5, and 6 dimensions in the bottleneck layer on four different datasets: American Sign Language (ASL) Gestures, Object Grasps, Activities of Daily Living (ADL) Tasks, and Combined. The performance is calculated using Variance Accounted For (VAF), which represents the difference between the output and the input. Solid lines represent average results across all paritcipants. Faint shaded lines represent 95% confidence interval. nAEN outperformed PCA across all datasets and for all number of latent dimensions tested in this study.

For all four datasets, nAEN outperformed PCA by reconstructing the input data with higher VAF for two, three, four, five, and six latent dimensions (Figure 5). The average VAF with just 2CUs across all datasets was 94% for nAEN whereas it was 78% with 2PCs for PCA.

Wilcoxon Signed-Rank Test revealed an effect on VAF by the dimensionality-reduction method (*p* < 0.001). When comparing nAEN and PCA, the difference in the performance between the two methods decreased as the number of dimensions in the latent manifold increased (Figure 5).

By performing the Friedman's Test on VAF produced by PCA and nAEN models with two-dimensional latent manifolds, an effect of datasets was revealed on both dimensionality-reduction methods (*p* < 0.001). Specifically, PCA performed similarly for both Object Grasps and ADL Tasks datasets (*p* = 0.986), but its performance decreased significantly for ASL Gestures (*p* = 0.002 and *p* = 0.006 when compared to Object Grasps and ADL Tasks, respectively). The nAEN, on the contrary, had higher performance for ASL Gestures over two dimensions in comparison to ADL Tasks (*p* < 0.001) and Combined (*p* < 0.001) datasets.

**Figure 6:**
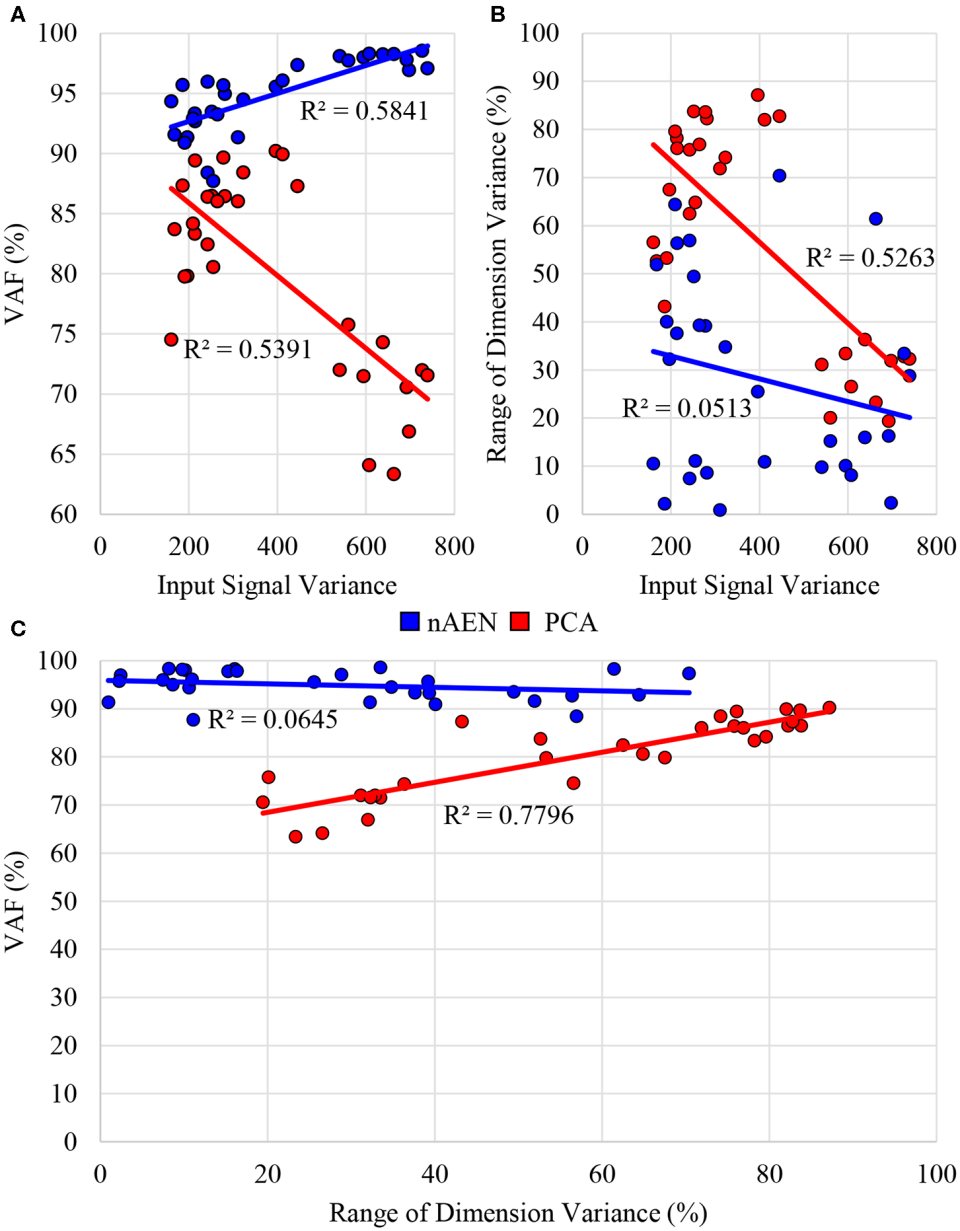
**(A)** Correlation between average signal variance of input data across 10 participants and Variance Accounted For (VAF) with two-dimensional latent manifold plotted for three different datasets (American Sign Language (ASL) Gestures, Object Grasps, Activities of Daily Living (ADL) Tasks) for non-linear Autoencoder Network (nAEN, blue) and Principal Component Analysis (PCA, red). **(B)** Correlation between average signal variance of input data across 10 participants and range of dimension variance for 2 Coding Units (CUs)/Principal Components (PCs). **(C)** Correlation between VAF and range of dimension variance for 2CUs/PCs for three datasets (ASL Gestures, Object Grasps, ADL Tasks). PCA exhibited some correlation across all three scenarios tested whereas nAEN experienced no correlation.

**Figure 7:**
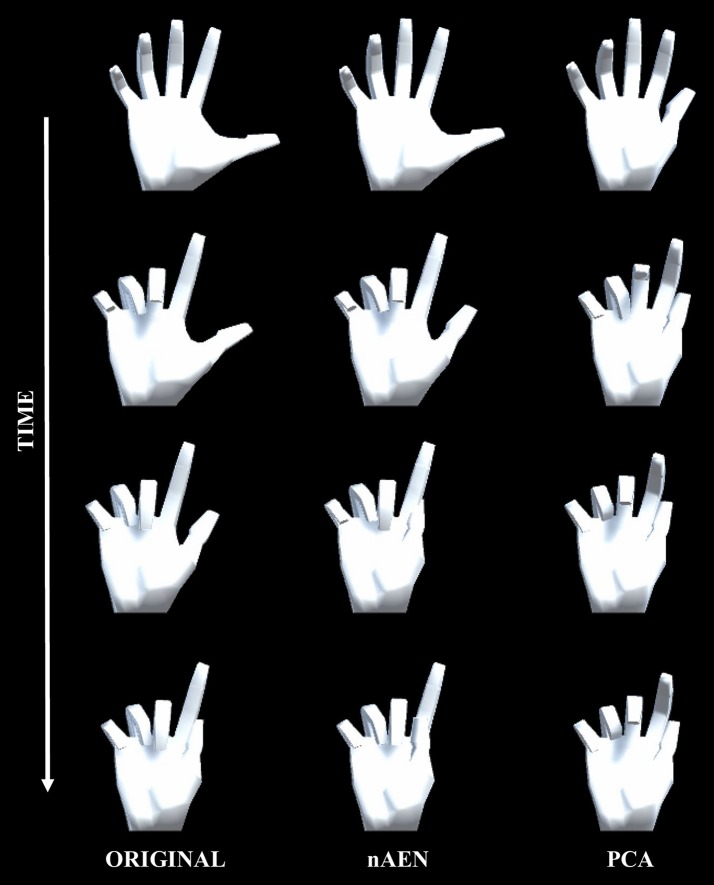
Visualization of gesture 1 from American Sign Language Gestures dataset, reconstructed from two-dimensional latent manifolds of non-linear Autoencoder Network (nAEN, middle column) and Principal Component Analysis (PCA, right column). The reconstructed gestures were compared to original gesture (left column). A few snapshots of each gesture were taken in time from REST to ACTIVE states.

Accordingly, the correlation between VAF and signal variance of each dataset was analyzed (Figure 6A). The *R*^2^ values for a linear model between two variables were 0.58 and 0.54 for nAEN and PCA, respectively. The relationship is positive for nAEN and negative for PCA (observed from the slopes of the linear regression lines). Lastly, from Figure 7, one can observe the visual differences in the performance of two dimensionality-reduction methods. While nAEN (middle column) was able to closely match the original hand gesture (left column), PCA (right column) failed to reconstruct the proper flexion of middle, ring, and pinky fingers as well as the full extension of the index finger. Reconstruction of all gestures and trials is shown in Supplementary Video.

### Dimension Variance

**Figure 8:**
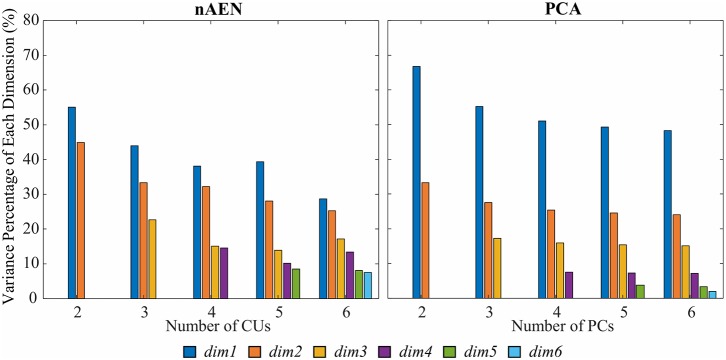
Variance percentage of each latent dimension (represented with different colors) with respect to the overall variance of the dimensions considered in the analysis [number of Principal Components (PCs)/Coding Units (CUs)] for P1 performing American Sign Language (ASL) Gestures. Two, three, four, five, and six dimensions in the bottleneck are compared for non-linear Autoencoder Network (nAEN) and Principal Component Analysis (PCA) and ranked by order of decreasing variance. nAEN exhibited smaller drop of variance across latent dimensions in comparison to PCA for P1.

Variance spread across nAEN and PCA dimensions was plotted for participant P1 performing ASL Gestures (Figure 8). Variance dropped significantly for each new added PC dimension whereas nAEN exhibited a less prominent decrease in variance for every additional latent dimension. The sharper drop in PCA variance appears to be a consequence of the orthogonality of subsequent eigenvectors.

**Figure 9:**
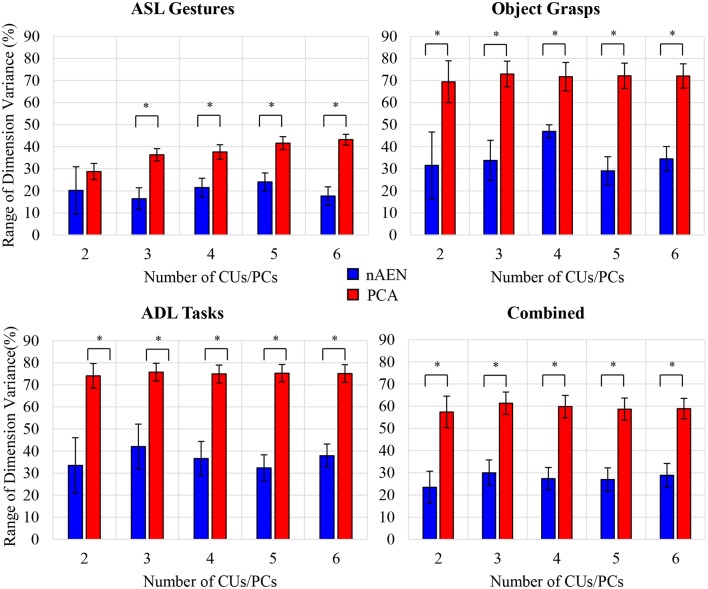
Difference between dimensions with highest and lowest variance values averaged across all participants for four datasets: American Sign Language (ASL) Gestures, Object Grasps, Activities of Daily Living (ADL) Tasks, and Combined. Statistical significance: *p* = 0.106 for ASL Gestures for 2CUs/PCs; *indicates statistical significance of *p* = 0.002. nAEN exhibited smaller drop of variance across latent dimensions in comparison to PCA across all datasets and for all number of latent dimensions except for 2 dimensions in ASL Gestures.

Such behavior was consistent across all participants, as described by the average difference between dimensions with the highest and lowest variance (Figure 9). This difference was greater for PCA than for nAEN and significant in all conditions (*p* = 0.002), with the exception of ASL Gestures reconstructed with 2CUs/PCs (*p* = 0.106), according to Wilcoxon Signed-Rank Test.

In addition, an interesting observation could be made in regard to the average difference between latent dimensions with highest and lowest variance across three datasets (ASL Gestures, Object Grasps, ADL Tasks). When plotting the range of dimension variance vs. the signal variance across each dataset, a correlation is revealed for the PCA case—*R*^2^ of 0.53 (Figure 6B). No such correlation was found for nAEN (*R*^2^ of 0.05). The relationship found for PCA was inverse: higher signal variance dataset resulted in lower difference between latent dimensions with highest and lowest variance.

Lastly, plotting the VAF of each participant for the three datasets vs. the range of dimension variance for 2CUs/PCs reveals similar correlation for PCA (*R*^2^ = 0.78) and no correlation for nAEN (*R*^2^ = 0.06) (Figure 6C).

### Latent Trajectories Visualization

**Figure 10:**
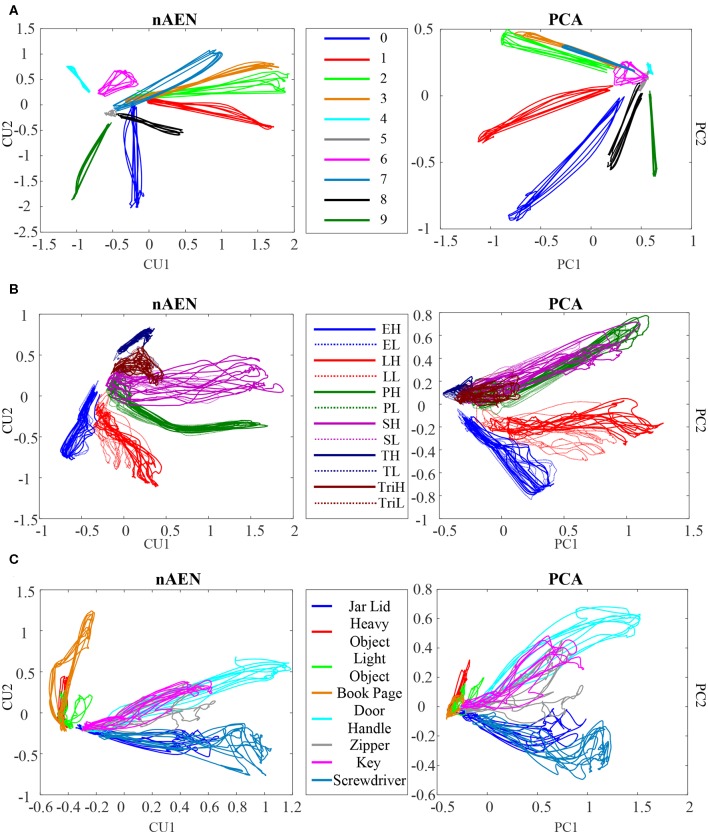
Visualization of the latent trajectories for nAEN and PCA for P1 performing **(A)** American Sign Language (ASL) Gestures, **(B)** Object Grasps, and **(C)** Activities of Daily Living (ADL) Tasks. The hand movements tested are represented with different colors. The latent manifold only consists of two dimensions. For Object Grasps, the legend can be interpreted as follows: the first letter represents the type of grasp (E, Extension; L, Lateral; P, Power; S, Spherical; T, Tip; Tri, Tripod); the last letter represents the weight of the object being grasped (H, Heavy; L, Light).

nAEN and PCA latent trajectories were visualized for 2CUs/PCs in the case of participant P1 performing ASL Gestures (Figure 10A), Object Grasps (Figure 10B), and ADL Tasks (Figure 10C). All trials used in the analysis were plotted, and separate gestures were indicated using different colors.

The overall structures of the plotted latent trajectories were similar across PCA and nAEN, with different gestures visually separated. Some movements appeared closer to each other in the 2D manifold than to other movements. Certain ASL gestures (e.g., gestures 2 and 3) appeared closer to each other than to others in both nAEN and PCA manifolds. In Object Grasps, heavy and light versions of the same grasp took the same part of the visualization space. ADL tasks that required similar type of grasping (e.g., using a screwdriver and opening a jar lid) appeared in the same part of the 2D space.

### Movement Separability

**Figure 11:**
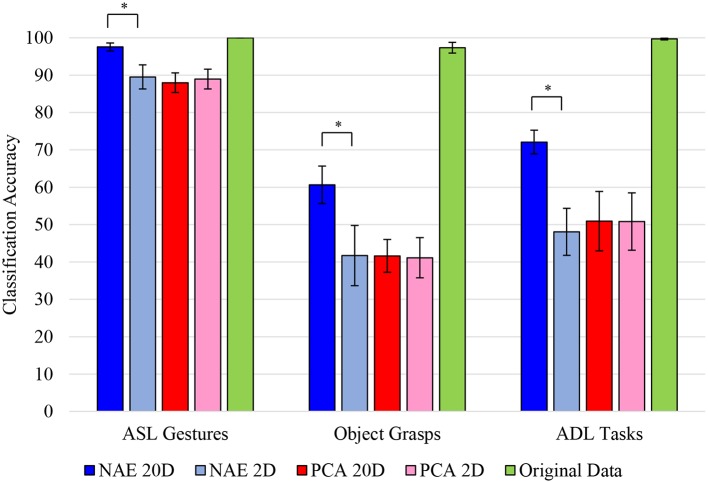
Accuracy of SoftMax regression applied to different datasets [American Sign Language (ASL) Gestures, Object Grasps, Activities of Daily Living (ADL) Tasks] across all participants. Regression was applied to original input data (green), reduced non-linear Autoencoder Network (nAEN) 2D (light blue) and reconstructed 20D (dark blue) data, as well as reduced Principal Component Analysis (PCA) 2D (light red) and reconstructed 20D (dark red) data. Error bars represent 95% confidence interval. Statistical significance: *indicates statistical significance of *p* < 0.001. No significant difference was found on the reduced 2D manifold of nAEN and PCA across all datasets. Original data exhibited high (nearly 100%) of movements. There was a significant difference between the reconstructed (20D) and reduced (2D) manifolds of nAEN with the former being more separable than the latter.

SoftMax regression on the 2D manifold of nAEN and PCA did not reveal any significant difference in separability of movements between the two methods (Figure 11). The difference was insignificant across all three datasets (*p* = 0.846 for ASL Gestures, *p* = 0.695 for Object Grasps, and *p* = 0.557 for ADL Tasks).

There was a significant difference (*p* = 0.002) in the classification accuracy when the 20D manifolds, reconstructed from two latent dimensions, were evaluated. Across all three datasets, nAEN generated more separable representations than PCA. There was no significant difference between the separability of the reconstructed 20D and reduced 2D representations with PCA across ASL Gestures (*p* = 0.695), Object Grasps (*p* = 0.492), and ADL Tasks (*p* = 0.695).

Lastly, when applying SoftMax regression on the original input 20D kinematic space, the separability of classes was higher than that of the reconstructed 20D and the reduced 2D manifolds for both PCA and nAEN. The classification accuracy was at nearly 100% across all datasets (Figure 11).

The overall separability was much higher for ASL Gestures than for Object Grasps or ADL Tasks datasets for the reduced 2D and reconstructed 20D spaces (Figure 11).

## Discussion

The complexity of a human hand makes the control of its prosthetic analog a challenging task. While posture control has been shown to be a novel and innovative way of providing a continuous controller for prosthetic users of their highly sophisticated devices, it has been limited by the linear nature of its underlying dimensionality-reduction algorithm. In this study, a non-linear equivalent of PCA, nAENs, demonstrated higher performance in: (i) reducing complex hand kinematics into a lower dimensional manifold with a smaller loss of data variability, (ii) creating higher spread of dimension variance in the latent manifold, and (iii) reconstructing a more separable manifold. All of these points could make nAEN a potentially effective at supporting continuous posture control for prosthetic hands.

### Dimensionality Reduction

The ability to reduce the dimensions of kinematic data without ignoring their effective complexity is an essential yet challenging task to understand the biological mechanisms of control as well as to design precise artificial controllers. When it comes to developing a controller for multi-DOF hand prostheses, reducing the number of control signals may result in a more intuitive interface for the user. While the human brain is able to simultaneously manage multiple DOFs, such as those in the hands, often with high dexterity and precision, many research studies suggest that it does so through a smaller number of control “knobs” identified as synergies (Santello et al., 1998; Todorov and Ghahramani's, 2004; Weiss and Flanders, 2004; Feldman and Latash, 2005; Ting and Macpherson, 2005).

To understand how dimensionality reduction may preserve the essential complexity of behavior, investigators have applied dimensionality-reduction techniques such as PCA to human hand coordination (Santello et al., 1998; Todorov and Ghahramani's, 2004). Santello's group determined that 2PCs were sufficient to account, on average, for ~84% of hand kinematic variance, a higher value than the figure in our study. Such difference may be explained by the difference in the kinematic data. While the entire range of kinematic data from the REST to ACTIVE positions was recorded and used for analysis in our study, Santello's group only utilized one static instance of kinematics per imaginary grasp. In addition, the number of signals that was used in the Santello et al. was 15 whereas our study utilized 20 kinematic signals for analysis, which could have made it more difficult for PCA to reduce the dimensionality of the kinematic space.

Such dependence of PCA performance on the number of analyzed signals was also presented in the study of Todorov and Ghahramani's (2004) as they ran PCA on 20 and 15 kinematic signals from a sensorized glove. They determined that in a 20-signal analysis, more PCs were required to account for the same variance of data than in a 15-signal analysis. In addition, they calculated that anywhere from three to seven PCs were required to account for 85% of data variance if the analyzed angle data was raw without normalization. The number of PCs depended on the performed task. In the aforementioned study by Todorov's group, the analysis was performed on a larger set of kinematic samples, similar to our study; however, it was done on individual tasks rather than full datasets containing various tasks.

In our study, it was also noted that PCA performance was significantly inferior for ASL Gestures than for Object Grasps and ADL Tasks with just 2PCs. Similar behavior could be observed in Todorov and Ghahramani's (2004) study where the number of PCs to describe individuated joint movements was higher than that for object grasping and manipulation. This might be explained by the kinematic complexity of ASL gestures, in which independent joint control is required. In ADL tasks and object grasping, on the contrary, many joints move simultaneously to achieve the desirable posture or grasp, potentially joining into linear combinations that can be easily detected by PCA.

It is interesting to note that while PCA appeared to be less efficient with the ASL Gestures dataset (reconstructing with lower VAF), nAEN exhibited stronger performance with the ASL dataset (reconstructing with higher VAF) than with Object Grasps or ADL Tasks. This point was also clearly indicated in the inverse relationship between the signal variance of a dataset and the VAF with 2CUs for nAEN. As a result, nAEN improved VAF and signal reconstruction when the input signal had more variability (as in ASL Gestures), contrary to the linear PCA. This is consistent with the observation that attempting to estimate the dimension of the data generated by a non-linear process with a linear method, like PCA, results in the overestimation of the actual process dimensionality (Tenenbaum et al., 2000). Accordingly, adding variance to the input data would result in the reduction of VAF by an insufficient number of PCs.

When comparing the performance of linear and non-linear dimensionality-reduction techniques, our study demonstrated that nAEN outperformed PCA by reconstructing over 90% of data variability with only 2CUs. Such results overpower the dimensionality-reduction performance of PCA presented in earlier hand kinematic studies. A comparison of non-linear dimensionality reduction was performed earlier by Romero et al. (2010) and by Cui and Visell (2014) over datasets obtained from hand grasping patterns. Somewhat surprisingly, Cui and Visell concluded that the quality of dimensionality reduction obtained by PCA was superior to that obtained by non-linear algorithms, including AEN. Our findings are not consistent with their conclusion. We believe that this discrepancy may be attributed to two factors. First, the analysis of Cui and Visell was limited to grasping, whereas our data set included other hand task. Perhaps, most notably, our data included ASL gestures and a broader spectrum of hand configurations associated with ADLs. In fact, in our dataset, the difference in performance between PCA and nAEN was smaller for hand grasps. A second observation concerns the performance measures. While we base our conclusions on VAF, Cui and Visell adopted a criterion based on the preservation of neighborhood relations after dimensionality reduction. This criterion was based on Euclidean distance, which, as noted by the authors, has an implicit bias in favor of a linear method like PCA. And one can add that Euclidean distance is not a clearly applicable measure for angular manifolds. Like Cui and Visell, Romero and colleagues limited their analysis to grasping patterns. They compared the latent manifold generated by different non-linear dimensionality-reduction algorithms observing a better performance compared with PCA.

Our findings highlight the potential superiority of nAEN when used as a control method for hand prostheses. Higher VAF value signifies that with a smaller number of control signals, the nAEN-based controller would be competent to generate a more precise representation of multiple DOFs in a prosthetic hand, compared to PCA. As a result, tasks that require high precision and dexterity (e.g., handcrafts, personal hygiene) may become more feasible. The decreased number of control signals required to control a precise motion of the prosthesis may result in lowering the mental burden on the users without sacrificing performance. For example, current techniques, such as PR in Coapt Gen2 (*Coapt LLC, Chicago, IL, USA*), rely on eight EMG signals to control a prosthetic device. The results of our study suggest that potentially by reducing the number of EMG signals to two and allowing each signal to control one CU would still allow the user to cover a large space of hand gestures.

The findings of this study also suggest the inherent non-linear nature of hand kinematics. The apparent differences between nAEN and PCA with only two dimensions in the latent manifold highlight the former's ability to capture components of the data that are not being picked up by the linear function of PCA. Evidently, this may suggest the need to, first, test and, potentially, utilize non-linear methods when analyzing biological systems, such as hand kinematics. While their mathematical manipulation might be more difficult and less intuitive, the importance of capturing non-linearities of a system might be of greater importance.

We feel it is important to emphasize that our use of an artificial neural network, the autoencoder, is not associated with any claim or pretense to represent information processing in the neural system. This is presumably a limit shared by PCA, as there is no evidence that constraint of orthonormality is satisfied by neuromuscular activity. Nevertheless, a relevant element in our analysis is that the information that can be extracted from the observation of hand motions is better captured by a low-dimensional non-linear manifold than by a linear space. While there are other non-linear statistical methods for signal processing (Tenenbaum et al., 2000), AENs have the distinctive property of acting as non-linear filters whose parameters are set by training on an initial dataset. Then the network with these same parameters is used to project incoming data on the same latent manifold of the initial dataset, under the critical assumption that the signal statistics has not changed.

### Dimension Variance

Dimension variance is an important aspect of every controller. In some cases, the distribution of control authority across the controlled dimensions should be uniform as controlling each DOF may be similarly important. One of such cases includes control of a two-dimensional cursor on a screen—in Euclidean geometry, where all directions are by definition equivalent, the control of each dimension should be distributed equally. However, if the dimensionality reduction results in an uneven distribution (a large range of dimension variance), then control is distributed unevenly.

While keeping the number of control signals as low as possible is important in ensuring the mental load to the user is manageable, adding more signals can be crucial to allow the controller to account for important information that may otherwise be thrown away. As a result, it might not be useful to limit the controller to the minimal number of control signals.

In such cases, the dimensionality-reduction method of choice may be detrimental. From the results presented above, nAEN could prove its feasibility as a method that would allow developers adding control signals that account for task-relevant variability. In the case of PCA, if the controller requires six signals, the last two signals oftentimes have the variance of <5% of the entire range of motion for all six dimensions. In most cases, such addition would be equivalent to adding noise. Noisy signals may decrease the controller performance and negatively affect the user's ability to perform tasks. In nAEN, on the contrary, adding more control signals does not organize data variance in a decreasing manner across dimensions but rather does so in a proportional manner where no signal accounts for <10% of data variability.

With a much smaller difference between the dimensions of highest and lowest variance for larger number of dimensions, nAEN highlights its ability to distribute data information across CUs more evenly than PCA.

Another interesting point that was made in the analysis is the correlation between input data variance and the variance spread across latent dimensions in PCA. It appears that data of higher variance (e.g., ASL Gestures) results in latent dimensions with a smaller range of variance, implying that variance is distributed more equally across dimension. On the contrary, when input data exhibits less variance (e.g., Object Grasps, ADL Tasks), there is a large difference between dimensions of highest and lowest variance. Similarly, when VAF is high, PCA appears to exhibit a more dramatic drop between dimension variance, thus proving this dimensionality-reduction method to be the less desirable choice even in cases when it exhibits high VAF. Such results highlight that in cases when PCA is able to reconstruct with a desirable VAF, the variance across its latent dimensions will not be equally spread, thus resulting in a higher chance of producing control signals that would appear noisier.

### Latent Trajectories Visualization

Visualization of the latent trajectories can be useful in understanding the internal works of a dimensionality-reduction method. In addition, if this latent structure is utilized in the controller, it can aid researchers in identifying the properties of the control manifold. While visualization of the latent trajectories of PCA is simple due to the orthonormality of the principal eigenvectors and the overall linearity of the space they span, things are different with nAEN since CUs are neither orthogonal nor linear elements.

As one visualizes the latent trajectories of a nAEN by representing CUs as Cartesian coordinates, one must understand that this representation is unable to capture the non-linear properties of the manifold. However, research in visualization of non-linear manifolds has been limited.

In this study, the 2D Cartesian representation of the latent trajectories was used to visualize the separability of different movements within each dataset. From the results, it appeared that there was no significant difference between the separability of classes of nAEN and PCA manifolds across all subjects.

When visualized, certain movements appeared to be much closer in the 2D manifold to some than to others, increasing their chance of being misclassified. Such spatial closeness could be explained by the kinematic similarity of certain movements. For example, gestures 2 and 3 were closely placed on the 2D manifold of both nAEN and PCA. When examining the two gestures kinematically, one could notice that the only difference between the two was in the flexion of the thumb. Likewise, movements that were very different from each other kinematically (e.g., gestures 1 and 9) appeared further away from each other on the 2D plot. Same results could be seen across other datasets, indicating that kinematic similarity resulted in closer appearance of the movements on the 2D manifold.

### Movement Separability

Separability between nAEN and PCA in 2D was participant- and movement-dependent, implying that some participants created a more separable nAEN manifold for certain tasks while others exhibited a more separable PCA manifold for the same tasks. Such occurrence may be due to the kinematic difference across participants performing various tasks. It is also important to note that the goal of the created nAEN structure was to minimize the error between the output and the input and not to create a more separable manifold of reduced dimension. However, one could rewrite the algorithm in such a way that the network would search for parameters that create a more separable manifold in the bottleneck layer.

It was noted in the results that separability of movements was much higher for ASL Gestures than other datasets for both nAEN and PCA. This could potentially be explained by hand kinematics being more distinct during ASL Gestures that clearly differentiate different classes. This is consistent with the very purpose of a sign language to generate readily distinguishable patterns. In addition, it is important to note that both heavy and light variations of the same grasp type were used as separate classes in Object Grasps, which could make it more difficult for the classifier to differentiate between them since they were very similar kinematically. If other information, such as for example, kinetic, was used in addition to kinematic data to differentiate between different grasps, the classification accuracy of Object Grasps would potentially be higher. Lastly, in ADL Tasks, many movements required similar grasping types, which, in turn, resulted in similar kinematic output, making classes less differentiable.

While nAEN exhibited an increase in separability of classes when going from the 2D latent manifold to its 20D embedding, no such difference was observed for PCA. The latter result is expected, because with PCA the latent manifold is a 2D plane embedded in the 20D dimensional signal space. In this linear case, the Euclidean distances between points in the plane are the same if we take them over the plane or over the embedding signal space. The same cannot be concluded with non-linear dimensionality reduction, because the latent manifold is now a curved space and distances between points over a curved surface are generally different when taken over the surface or over the embedding space. In fact, distances over a curved surface (think of a sphere) can only be longer than the differences over the embedding signal space. The results in Figure 10 show that this difference in Euclidean metric leads to a poorer classification when the distances are taken in the local coordinates of the latent manifold, as points belonging to the same class are mislabeled as belonging to different classes. Figure 10 also shows that there is not a difference in classification accuracy between nAEN and PCA, when the data are taken in the respective low-dimensional latent representations. Therefore, we do not have a case for using the non-linear rather than linear dimensionality reduction for a prosthetic controller based on PR. However, the conclusions are different for a prosthetic system based on continuous control, where the reconstruction error and the variance accounted for play a greater role and where these both best captured by the non-linear dimensionality reduction (Figures 5, 6A).

An interesting note could be made regarding the high separability of classes in the original input data. It is important to understand that the original data contains 20 signals that can each vary across different movements. And although every movement started from approximately the same position in every dataset, differences in signals that could appear insignificant on their own could result in a significant difference when added together across all 20 signals. Hence, it is intuitive that classes are more separable when more dimensions are present. However, in such a case, both PCA- and nAEN-reconstructed 20D spaces should exhibit just as high accuracy. While we noted before that the PCA-reconstructed space might not see a significant improvement in class separability when going from 2D to 20D, a careful consideration of the nAEN results must be made. Although there was a significant improvement when going from 2D to 20D for nAEN, the 20D results were still not as high in accuracy as those of the original input data. This could be explained by the potential elimination of insignificant signal differences during reconstruction by the nAEN. These insignificant differences could be taken by the autoencoder as noise, which it aimed to reduce in the system, only leaving information that produced high variability. As a result, data with small variability is not reconstructed. This observation can also lead to the conclusion that PCA, when reconstructing, removes more of low-variability samples from the data, resulting in a less separable reconstructed space.

### Other Applications in Prosthetic Control

It is important to mention that the findings of this study might have wider applications beyond the myoelectric control. One of such examples would be in hardware development similar to the aforementioned “Soft Hand,” in which the simplified prosthetic control relies on the linear combination of the first *n* PCs (Della Santina et al., 2017). This results from the linear nature of PCA that is discussed earlier in this paper. To obtain the desired posture in a hardware using nAEN, one might utilize the *decoder* part of the network and pass the first *n* CUs through the third and fourth layer as described in Equations (3) and (4). In such way, the development of the hardware of the prosthetic hand would rely on the composition of the decoder component of nAEN.

In addition, when dealing with myoelectric control for prosthetic hands, one must consider not only the domain of kinematic behaviors of the device, but the control signals themselves (i.e., EMG). While reducing the dimensionality of kinematics is of high importance, one can consider applying dimensionality-reduction methods studied in this paper to the control signals. This would expand the potential of using a greater number of signals, fused in a latent manifold, for prosthetic control. Such application of nAEN will be explored with EMG signals in future research of improving myoelectric control of hand prosthesis.

### Supervised vs. Unsupervised Learning

Many of the state-of-the-art techniques in prosthetic control involve supervised learning methods. For example, the PR method utilizes a form of a classification algorithm, which typically consists of a feature extraction from the given EMG signal and feature classification of the desired hand movement (Geethanjali, 2016). With the use of this technique, prosthetic users are able to associate certain EMG patterns with desired grips, thus decreasing the time it takes to select and perform the anticipated movement. Despite of its rising popularity, PR control exhibits issues such as long training time, chances of inaccurate classification, and being limited to a finite number of preselected hand postures (Hargrove et al., 2006; Scheme and Englehart, 2011; Young et al., 2011; Castellini et al., 2014; Atzori et al., 2016; Geethanjali, 2016).

In addition, all forms of supervised learning are limited by their dependence on labeled data, the ground truth, based on which they learn a function that best approximates the relationship between the input and output observable in the data. For unimpaired individuals, creating supervised output might not be an issue (e.g., flex a joint to a specific degree, etc.). However, for individuals with motor impairments, such task is inherently difficult or completely unachievable, making the creation of labeled data impossible.

In contrast, PCA and AENs are unsupervised algorithms, whose data do not need to be labeled as their goal is to learn the data's statistical properties rather than minimizing some classification error. As a consequence, a user interface based on unsupervised methods can adapt to the particular statistics (e.g., in kinematics) of the users without requiring them to perform specific movements in specific ways.

Another form of supervised learning is regression, which some research groups have proposed for prosthetic control (Muceli and Farina, 2011; Muceli et al., 2013; Ngeo et al., 2014; Geethanjali, 2016). This is a form of a learning method, in which the output data is continuous in contrast to that of a classifier. While it may be a useful feature in the development of a continuous controller, in which device movements are not limited to a number of preset postures, regression has not been proven effective in its use with prostheses. Oftentimes, regression algorithms developed for prosthetic hands require clean forearm EMG signals that can be associated with certain hand movement. However, in case of transradial (below-elbow) amputations, these signals are heavily dependent on the site of amputation, amount of residual limb, and many other factors (Li et al., 2010). Clean surface EMG signals can be obtained in a lab setting from able-bodied individuals, but are much harder to get from amputees outside of the lab, thus making regression less effective control tool for prostheses.

In summary, unsupervised continuous learning methods, such as nAENs, promise to be a useful tool in the development of prosthetic controllers in addition to their superior performance in dimensionality reduction.

## Data Availability Statement

The raw data supporting the conclusions of this article will be made available by the authors, without undue reservation, to any qualified researcher.

## Ethics Statement

The study involving human participants was reviewed and approved by University of Washington's Institutional Review Board. The patients/participants provided their written informed consent to participate in this study.

## Author Contributions

AP-F contributed to design and implementation of the study as well as data analysis performed for the experiment. AP-F also wrote the manuscript with the support of FR, ER, IN, MC, and FM-I. FR contributed to design of the neural network architecture described in the paper, providing necessary code for certain parts of data analysis, and educating on pre-processing steps. ER and FM-I contributed to conception and design of the study as well as interpretation of the results. All authors contributed to manuscript revision, read, and approved the submitted statement.

## Conflict of Interest

The authors declare that the research was conducted in the absence of any commercial or financial relationships that could be construed as a potential conflict of interest.

## Supplementary Material

Supplementary Video Reconstruction of American Sign Language (ASL) gestures from two-dimensional latent manifolds of non-linear Autoencoder Network (nAEN, middle column) and Principal Component Analysis (PCA, right column). The reconstructed gestures are compared to original gesture (left column). Each gesture is presented and reconstructed five times (five trials).
